# "There’s Not Enough Bodies to Do the Demand": An Exploration of Key Stakeholder Views on the Role of Health Service Capacity in Shaping Cancer Outcomes in 7 International Cancer Benchmarking Partnership Countries

**DOI:** 10.34172/ijhpm.2020.254

**Published:** 2020-12-22

**Authors:** Maureen Seguin, Melanie Morris, Martin McKee, Ellen Nolte

**Affiliations:** Department of Health Services Research & Policy, London School of Hygiene & Topical Medicine, London, UK.

**Keywords:** Health System Capacity, Cancer Care, Cancer Outcomes, High-Income Countries

## Abstract

**Background:** Differences in cancer survival are shaped by differences in health system capacity in workforce and infrastructure. Part of the International Cancer Benchmarking Partnership (ICBP), this study explored stakeholders’ perceptions of the role of health system capacity necessary for cancer care in influencing cancer survival in 7 high-income countries.

**Methods:** We conducted semi-structured interviews with 79 key informants from national, regional, and local tiers of health systems, professional bodies, patient associations, and academic experts in Australia, Canada, Denmark, Ireland, New Zealand, Norway, and the United Kingdom. Data collection was guided by a conceptual model linking characteristics of health systems and cancer survival along the cancer patient journey, from recognition of symptoms at pre-diagnostic stages through to survivorship or death. Data were analysed using a thematic approach.

**Results:** We identified 3 themes as important in shaping cancer outcomes: primary care and access to diagnostic evaluation, specialist care and access to treatment, and workforce pertaining to diagnostic and treatment phases. Improved infrastructure for diagnosis and treatment had improved cancer outcomes in all jurisdictions. However, this was seen as insufficient if staffing was inadequate. Consolidation of services and greater surgical specialisation was important in some jurisdictions if accompanied by a reconfiguration of services, in particular the creation of specialist multidisciplinary teams, along with supporting capacity in the wider health system. Staff shortages were commonly cited as reasons why some jurisdictions lagged behind others.

**Conclusion:** Continued improvement in cancer outcomes will require sustained investment in plans to deliver and maintain the workforce engaged in cancer care and in the infrastructure on which they depend. However, strategic plans must recognise that systems for cancer care do not work in isolation from the rest of the health system and a whole systems approach is essential if we are to improve outcomes for an ageing, increasingly multimorbid population.

## Background

**Key Messages**

** Implications for policy makers**

- International variation in cancer survival is widely attributed to differences in the relative effectiveness of healthcare but it remains unclear what health system features shape cancer care in a way that can improve outcomes.
- This study highlights the complexity of factors that contribute to observed improvements in cancer survival across countries and over time. Although the relative importance of different factors varies across settings, we find that investment in resources, in the form of staff, infrastructure, and equipment for diagnosis and treatment, and processes, in particular service consolidation and surgical specialisation, are linked to improved quality of care.
- Continued improvement in cancer outcomes will require sustained strategic investment in plans to deliver and maintain the workforce engaged in cancer care and in the infrastructure on which they depend.
- Strategic plans must recognise that systems for cancer care do not work in isolation from the rest of the health system and a whole systems approach is essential if we are to improve outcomes for an ageing, increasingly multimorbid population.

** Implications for the public**

Countries worldwide are investing resources to make it more likely that people with cancer will be diagnosed early, access timely treatment, and increase their chances of surviving. Yet they vary in their degree of commitment, as do cancer survival rates among their populations. Even countries that have otherwise similar health systems differ in the way they have built and sustained the ability to deliver effective cancer care. Investments are often short term or narrowly focused, for example on equipment for diagnosis or treatment, without the staff needed to operate them. Our research stresses the importance of investing in the entire range of components of an effective service for patients with cancer.

Cancer survival is widely used as an indicator of the quality of health systems, with differences in survival between countries, and regions within countries, attributed to differences in the relative effectiveness of healthcare.^1-6^ Increasingly, research has sought to understand how specific health system features may be linked to cancer outcomes and explain the widespread variation in survival. Health system features have thus far mostly been considered under broad headings, such as spending on cancer care or healthcare in general,^7,8^ focused on sub-systems, such as the role of primary care in providing access to timely diagnosis,^9-12^ or investigated specific indicators of access, for example, to cancer drugs,^10,13-15^ or a combination thereof.

 Yet, all of these features are part of the wider health system, which depends on a range of inputs to function, such as health workers and physical capital (facilities and equipment). Although the performance of health systems ultimately depends on “the knowledge, skills and motivation of the people responsible for delivering services”^16^ [p. 77] the health workforce cannot deliver services effectively without the necessary infrastructure. In the cancer field, published evidence has tended to focus on the strength of individual specialties, such as medical or clinical oncology,^17-21^ and, more recently, the workload of medical oncologists,^22,23^ or the availability of equipment, for example, radiotherapy.^24^ These studies provide important insights into the capacity of cancer care systems, but, to our knowledge, only a small number of studies, such as those undertaken by the Örenäs Research Group,^25,26^ have examined the role of the workforce and physical infrastructure, and their interdependencies, in shaping cancer outcomes. This study contributes to filling this gap.

 The analyses presented in this paper are part of a wider study situated within phase 2 of the International Cancer Benchmarking Partnership (ICBP-2), a network of policy-makers, academics and clinicians researching factors contributing to differential cancer outcomes among countries.^27^ ICBP-2 includes 7 countries: Australia, Canada, Denmark, Ireland, New Zealand, Norway and the United Kingdom, chosen because they all have high-quality population-based cancer registries, provide universal access to healthcare funded primarily from general taxation, and spend comparable shares of national income on health.^6^

 Throughout this paper, we use the umbrella term ‘capacity,’ broadly defined as the structures, processes and management systems that enable organisations and systems to function effectively.^28^ It includes human, physical, and knowledge resources and the processes used to transform these resources into services or processes. We focus on 2 core resources, namely workforce and infrastructure (facilities, equipment), examining their perceived role and their interdependencies along the cancer care journey, and how those involved in cancer care and policy believe that they influence differences in cancer outcomes among countries and over time.

## Methods

 We employed a descriptive qualitative research design,^29^ adopting an implicitly positivist approach, using interview accounts to understand what key stakeholders understood to have been the key policies, strategies or actions that helped (or hindered) improvements in cancer survival. Specifically, we conducted semi-structured interviews with key informants in a subset of the 21 jurisdictions included in ICBP-2. These were 2 of 3 Australian states and 3 of 10 Canadian provinces, along with Denmark, Ireland, Norway, New Zealand, and the 4 countries of the United Kingdom (n = 13 jurisdictions). The subset was chosen to represent a spread of size of jurisdictions and cancer survival estimates while keeping the number of interviews manageable within the limited time frame of the study. For Canada, we selected Ontario (most populous province,^30^ higher than average cancer survival for Canada^2^), Manitoba (mainly rural, average survival), and Nova Scotia (maritime; lower than average survival). For Australia, we selected New South Wales (high population density,^31^ lower than average survival for Australia) and Western Australia (low population density, higher than average survival). Our approach to the study was guided by a conceptual model that visualises the range of possible interrelationships and causal pathways linking health system factors and cancer survival along the cancer care journey, from recognition of symptoms at pre-diagnostic stages through to survivorship or death. We have described the model in detail elsewhere.^32^ In brief, it provides a first step in the identification of key factors that might be amenable to system-level interventions to enhance cancer outcomes internationally. It also served to focus our qualitative enquiry.

###  Participant Recruitment

 We sought to interview decision-makers at the different tiers of the system: hospital managers, regional authorities where relevant, and government officials (including arms’ length bodies), representatives of professional bodies and of patient associations, and other relevant agencies and bodies, both within the cancer field and with wider health policy expertise. We sought to recruit sufficient numbers of participants from each category of decision-maker to ensure data saturation. Key informants were identified through a combination of purposive and ‘snowball’ strategies,^33,34^ working with local ICBP-2 collaborators in the first instance as well as conducting systematic searches of websites of organisations representing the range of stakeholders listed above. Members of the ICBP Programme Board were not considered for interview. Where appropriate, we invited further study participants following recommendations of initial key informants.

 For each jurisdiction we sought representation from across the main stakeholder groups. The jurisdictions covered by ICBP-2 vary in population size and the way health services, and cancer services within them, are organised and governed. Hence, the range of experts to be interviewed varied by jurisdiction. Furthermore, states in Australia, provinces in Canada, and, to a lesser degree, the 4 UK countries, are subject to national (UK) or federal (Australia, Canada) policies and regulations; we therefore also included national level representatives from these countries. Informed by our previous work,^35,36^ we sought to recruit approximately 8 experts in Australia, 20 experts across the 4 jurisdictions in Canada, between 6 and 8 experts each in Denmark, Ireland, New Zealand and Norway and approximately 20-22 experts across the 4 countries of the United Kingdom.

###  Interviews

 Potential participants were contacted by email and invited to take part in an interview using telephone or the online telecommunications applications Skype or Zoom; they were provided with a project information sheet with the invitation. A maximum of 3 emails were sent before replacing a potential participant with an alternate. After agreeing to participate, the interview was scheduled and participants were provided with the topic guide and consent form.

 The topic guide was informed by the conceptual model, focusing on the diagnostic and treatment/management part of the cancer care journey. Interview topics explored stakeholders’ views on and understandings of cancer policies and services in their jurisdictions, specifically: likely explanations for observed improvements and variations in cancer survival; key achievements and (continued) challenges in organisation and delivery of cancer services along the care journey; and perceived effectiveness of policy levers and instruments driving cancer services and policies. To inform conversations, the topic guide included an illustration of survival trends in each of the 7 countries for the period 1995-2014 and for 4 cancer sites: oesophagus, rectum, pancreas and ovary, selected to represent different levels of survival between the countries; the topic guide is shown in Supplementary file 1. The selection of cancer sites and choice of observation period reflects the focus of ICBP-2. We also provided an illustration of the diagnostic and treatment part of the cancer care journey to facilitate discussions of enablers and barriers in the organisation and delivery of cancer services in participants’ jurisdictions (Figure). The interview guide was pilot tested with 3 members of the ICBP-2 programme board (representing 3 different jurisdictions) to assess understanding and applicability of the topics to different contexts.

**Figure:**
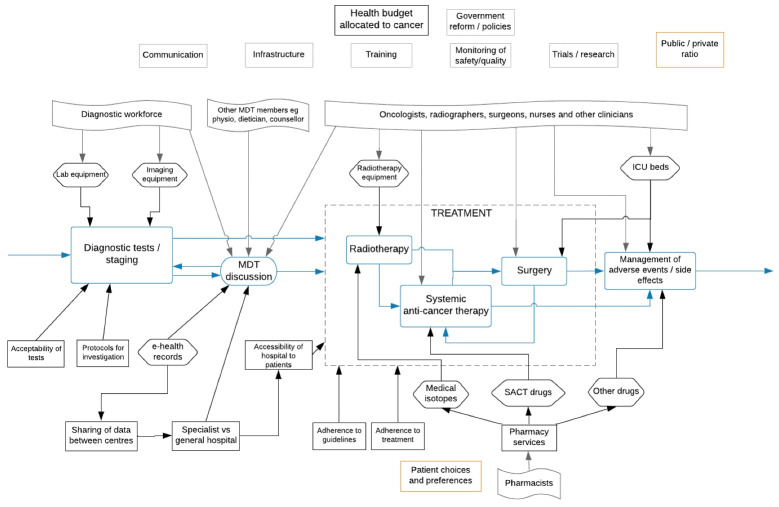
The Diagnostic and Treatment/Management Part of the Cancer Care Journey. Abbreviations: SACT, systemic anti-cancer therapy; ICU, intensive care unit; MDT, multidisciplinary team. *Source*: Adapted from Morris et al.^32^

 Interviews were conducted in English by 3 authors (MA, MMo, EN) from January 2019 to March 2020. Although we did not specifically exclude non-English speaking participants, all participants were fluent in English. The first 20 interviews were conducted in pairs of 2 to ensure consistency across interviewers. Six interviews included 2 or more interview participants. Interviews were recorded with participants’ consent and transcribed verbatim. Two interviews could not be recorded, and notes were taken instead.

###  Analysis

 Data analysis followed a thematic approach, using the stages of data familiarisation, coding (using NVivo version 12), and theme refinement. Our approach to analysis was largely deductive, guided by the World Health Organization (WHO) health systems framework^16^ and the framework developed by Atun et al for the analysis of national cancer control programmes.^37^ Specifically, we used thematic content analysis, which involved coding interviews using initial codes generated from the interview topic guide and the frameworks and developing new codes where data did not fit easily into existing categories. Following the coding of each interview, codes were collated to generate broader themes.

## Results

 We interviewed a total of 79 key informants in 13 jurisdictions (Table). Interviews varied in duration from 35 to 77 minutes, with an average of 54 minutes. Data saturation was achieved for each theme presented below.

**Table:** Organisations Represented by Interview Participants, by Country

**Jurisdiction**	**Type of Organisation (Main Role)**						
	**National/Regional Government **	**Regional/Local Hospital Management**	**Professional Association**	**Patient Organisation**	**Academia**	**Other** ^a^	**Total**
Australia^b^	5	2	1	1	1	1	11
Canada^c^	8	7	2	1	1	-	19
Denmark	1	1	1	2	1	1	7
Ireland	2	1	1	-	1	1	6
New Zealand	3	-	1	-	1	2	7
Norway	1	2	-	-	1	1	5
UK^d^	7	6	2	3	1	5	24
Total	27	19	8	7	7	11	79

^a^ For example cancer societies, charities, members of task forces or committees; ^b^ Australia, Western Australia, New South Wales; ^c^ Canada, Manitoba, Ontario, Nova Scotia; ^d^England, Northern Ireland, Scotland, Wales.

 We identified 3 themes as important in shaping cancer outcomes: primary care and access to diagnostic evaluation, specialist care and access to treatment, and workforce pertaining to diagnostic and treatment phases. We discuss each of these in turn.

 It is important to note that the majority of interview participants highlighted the complexity of factors involved that may have contributed to observed improvements in cancer survival over time in all jurisdictions. This was seen to be particularly relevant for the cancer sites explored in the interviews, for which it was not possible to isolate a single factor that would stand out in explaining trends, although there was widespread agreement that timely diagnosis was key to cancer survival. Some interview participants pointed to the interdependencies along the cancer pathway, with disruption of one factor impacting on the next, and, ultimately, affecting survival, which one participant referred to as *“the incremental effect of one roadblock on another roadblock on another one, on another one, on another one […]”* [R79 England]. We will come back to these interdependencies, and consequent knock-on effects, below.

###  Primary Care and Access to Diagnostic Evaluation

 Better access to timely diagnosis was widely perceived to have been an important contributor to observed improvements in cancer survival. This was attributed to a combination of increased investment in diagnostic imaging in most jurisdictions, particularly in the 2000s, both in terms of infrastructure and workforce, as well as greater standardisation of referral pathways to help reduce wait times for diagnostic investigations and, thus, treatment. Expanding diagnostic technology was referenced by several key informants from Denmark, so that they now have the highest number of PET (positron emission tomography) scanners in Organisation for Economic Co-operation and Development (OECD) countries (at 7.6 per million population in 2018), along with Australia (at 3.8 compared with 1.9 in Ireland, 1.4 in Canada, and 1 in New Zealand^38^), an observation that several participants from the United Kingdom also commented on (“*there is a general feeling that [Denmark has] moved faster than we have”* [R61 England]). Investment in other diagnostic equipment was also recognised as an important factor in survival improvements in other jurisdictions, as for example reported for Scotland:

*“[T]hey pour the money into endoscopic services to try and improve upper and lower GI [gastro-intestinal] endoscopy services, and other things to try and improve the diagnostic process and speed up the diagnostic process, so I think these are the sort of things that have helped” *[R76 Scotland].

 However, while diagnostic infrastructure and equipment were seen as necessary, increasing availability of such services in itself was viewed as insufficient to improve cancer diagnosis care. Accompanying measures to enhance skills and otherwise develop the workforce were essential, as well as greater standardisation of diagnostic pathways. Participants from several jurisdictions referred to the introduction of guidelines and standards to streamline referral from primary care to diagnostic evaluation, such as the urgent referral pathways for people with suspected cancer (‘2-week-wait’) in England from 1999, or fast track referral within the Danish cancer patient pathways in 2008, which had been found to reduce diagnostic intervals in those jurisdictions.^39,40^ Despite reducing wait times for those with ‘alarm symptoms,’ several participants highlighted the challenges of managing those with non-specific symptoms *“where it’s a small percentage probability for having cancer, then you often wait for too long” *[R68 Norway].

####  Ease and Speed of Referral

 In 2012, Denmark introduced urgent referral for nonspecific, serious symptoms via newly established diagnostic centres, and, more recently, so-called no-yes-clinics, in which the general practitioner (GP) has direct access to rapid investigations.^41^ Taken together, these developments were reported to have changed the approach taken by GPs, encouraging them to refer more patients:

*“So, if you have a GP that says that 50% of the patients that I sent in the cancer package [CCP] they do have cancer then you could argue he is very good in selecting patients, or you could argue he is really sending too few. […] And I think everybody agrees he’s really sending too few. So, he is missing a lot of cancer because he’s not sending enough” *[R22 Denmark].

 A recent shift in willingness of GPs to refer patients with vague symptoms for scans was also noted in Wales, supported by guidance by the 2005 National Institute for Health and Clinical Excellence^42^; this was expected to improve outcomes over time.

 Key to these changes is the ability to refer quickly. Danish participants identified the regionalisation of diagnostic services, with rapid diagnostic centres to facilitate diagnosis as contributing to improved cancer outcomes. Ireland introduced rapid access clinics from 2008 (for breast cancer, from 2014 for lung and prostate^43^), which respondents associated with earlier diagnosis and timeliness in initiating treatment. More rapid access to diagnostic testing was also seen as an important enabler in Australia, where GPs are empowered to order tests directly (rather than through a specialist), speeding up provisional diagnoses. Some in Australia cited this as contributing to Australia’s good performance in cancer outcomes relative to other ICBP countries (“*that’s the other issue that’s worth mentioning, that is different, is that a person who presents with a lump, for example, can go to their GP; their GP can actually then have a diagnostic phase, and go through that process, confirm the diagnosis of cancer, and then refer someone into the tertiary system”* [R58 Western Australia]). Their views were echoed by participants in New Zealand, who contrasted the Australian initiative with their experience of patients seeking private diagnostic testing there in order to circumvent delays at home.

 The ability to refer quickly (or to refer at all) is crucial and many jurisdictions reported how, despite improvements in diagnostic capacity over time, important challenges remained. One was rising demand for imaging, both cancer and non-cancer-related, with diagnostic services struggling to keep up. This was reported to be particularly problematic in those jurisdictions that historically had a lower capacity for diagnostic imaging, such as New Zealand (*“that whole system [complex imaging] is grossly overloaded”* [R30 New Zealand]), several provinces in Canada, and the 4 UK countries.

 Smaller jurisdictions found this especially challenging with, for example, 1 respondent from Canada reporting that while there is an expectation of one PET scanner for every 500 000 people, their province had only 1 for a population of 1.3 million, which *“basically means we’re always one little blown fuse away from not having a PET scanner”* [R15 Manitoba]. Respondents from Wales and Northern Ireland noted similar difficulties, with the latter highlighting the need to send patients to the Republic of Ireland or to England to access PET scans. Irish, Canadian and New Zealand participants commented that lack of scanning equipment had restricted scans to urgent or sicker patients. As a result, patients whose cancers presented with vague symptoms risked delay in diagnosis, something an Irish key informant linked to their poor survival with ovarian cancer.

####  Diagnostic Infrastructure

 Lack of strategic investment was cited by several respondents in UK countries (*“[F] unny thing that the NHS [National Health Service] is 70 years old and has never had a diagnostic strategy, and diagnosis […] is rather an important part of healthcare”* [R61 England]), with recognition that the United Kingdom was *“right at the bottom of the OECD country access rates to MRI and CT scanning”* [R62 England]. Access to capital funding was seen to be crucial in several jurisdictions, adding to the challenge of maintaining existing and replacing dated infrastructure, which was noted as a continuing challenge in Ireland and England especially:

*“So, really you have a backlog of infrastructural needs and that extends from the building to the equipment. Now Ireland already had quite an ageing capital stock coming into the [financial crisis of 2007/08] so we hadn’t really done much on capital investment” *[R16 Ireland].

 Clearly, it is important to have adequate diagnostic infrastructure but also the staff to operate equipment, an issue raised as a problem in many jurisdictions, especially among key informants based in Canada, Ireland, New Zealand, and the 4 UK nations, but also recognised as posing increasing difficulty in Denmark. Thus, even where equipment was available, shortages of key workers to operate scanners and interpret results limited access to diagnostic imaging. Shortages especially affected radiologists, endoscopists, and pathologists:

*“ So I think it’s pretty clear that diagnostic interval is a real concern and that’s largely down to workforce rather than necessarily equipment in Scotland. And that’s a problem that’s just been slowly getting worse over the years. […] the problem is, we don’t know the exact nature of those gaps and where they are” *[R66 Scotland].

 While these factors in themselves were widely viewed as compromising the diagnostic pathway, and ultimately affecting cancer outcomes, several key informants highlighted how blocks at one point in the diagnostic pathway had knock-on effects. Thus, respondents from both England and Wales reflected on the impacts that pressures on hospital capacity had on the ability of GPs to refer patients to these services, noting that they would be less likely to send their patients for a scan because they would not expect these to be carried out: *“unless we get a change of mindset there, and a change of improving capacity and putting the budget in for that, I’m not sure we will ever get to where Denmark and Australia are heading to”* [R61 England].

###  Specialist Care and Access to Treatment 

 There was general agreement among key informants across all jurisdictions that the treatment part of the cancer care journey had made important contributions to observed improvements, in some cases perhaps more so than improvements in diagnostics (*“once you actually get diagnosed, we are much more organised. We’ve got clear pathways, we’ve got the multidisciplinary working, all those things, follow-up pathways, guidelines, you name it, coming out of our ears*” [R77 Scotland]).

 Service consolidation and increased surgical specialisation were identified as crucial, especially for the cancer sites explored in interviews. Respondents from most jurisdictions commented on the important role that concentration of surgery for ovarian cancer in a smaller number of specialised centres had played in improving survival over the past 2 decades. Several key informants cited evidence of substantial reductions in numbers of facilities, with one respondent from Denmark noting how 50 hospital departments performing surgery in the late 1990s had fallen to 4 at the time of the interview. In Scotland, the ‘CRAG Report’ (Clinical Resource and Audit Group) on the management of ovarian cancer (1998)^44^ had led to the creation of managed cancer networks and the consolidation of surgery for ovarian cancer (*“within 5 years, instead of having something like 45 or 50 gynaecologists operating on ovarian cancer in Scotland, we essentially brought it down to a core of about 10 or 12”* [R76 Scotland]).

 Centralisation of surgery was linked to subspecialisation of surgeons, which some participants viewed as having contributed to improved outcomes. For instance, a respondent from Ireland who had worked in Ontario commented on how centralisation of care in the former had allowed surgeons to concentrate their scope of work (with thoracic/upper bowel surgeons operating exclusively on oesophageal cancer, and lower bowel surgeons on rectal cancer). They contrasted this experience to working in Ireland, where this process had moved much more slowly. This was echoed by another Irish respondent, who noted that surgical oncology, as a surgical subspecialty, was yet to emerge as a specific training stream. A respondent from Australia suggested that resistance among gynaecologists to embrace gynae-oncologists had delayed centralising ovarian cancer surgery, which, they argued, might explain why ovarian cancer outcomes *“are not where they should be compared to other cancers”* [R51 Australia].

####  Wider Service Reconfigurations

 While centralisation of surgery was clearly seen as important, there was recognition that on its own it was insufficient to explain improvements in survival. One key informant from Nova Scotia, Canada, noted that even though surgery for gynaecological and oesophageal cancer was centralised, survival was less than optimal. Participants from several jurisdictions highlighted the importance of ensuring that centralisation was accompanied by wider service reconfigurations, in particular the introduction of specialist and multidisciplinary teams (*“cancer treatment is a team sport” *[R10 Ireland]) and the co-location of resources, for example in designated cancer centres (eg, Ireland, Northern Ireland, Ontario).

*“So, the Cancer and Palliative Care Network did identify in the early 2000s a concern about outcomes for people with upper GI cancers, where surgery was happening at low volume centres, and effectively, worked very hard to determine what was a reasonable standard of accreditation for a centre to be able to undertake upper GI cancer surgery. And in doing so, I think substantially improve the survival of that group of patients, because of making sure that they’re tertiary hospitals that are well set up in terms of access to intensive care, specialised anaesthetics, etc, but also surgeons doing those procedures frequently, and with a high level of skill” *[R58 Western Australia].

 In England, respondents acknowledged the key role that the 2000 NHS Cancer Plan^45^ had played, for a range of malignancies, especially the supporting Improving Outcomes Guidance:^46^

*“[T] he decision was made […] that centres involving surgery should have a population of between 1 and 2 million people minimum to deal with oesophageal and gastric cancer, and between 2 and 4 million to offer surgery for pancreatic cancer. And that’s what happened, and it was not rigidly enforced across the United Kingdom, but it broadly did occur, and it’s occurred in most places” *[R79 England].

 These developments were accompanied by significant investments in radiotherapy in many jurisdictions, which participants identified as contributing to improving cancer outcomes. Similar to what we heard about diagnostic equipment and infrastructure, Denmark substantially increased its capacity: *“[A]bout 20 years ago […] we estimated the number of accelerators needed to deliver high standard cancer treatment. And now Denmark is one of the countries in the world with the highest concentration of accelerators”* [R01 Denmark]^[1]^. Sizable increases in radiotherapy capacity were also reported by respondents from Australia, Norway, Ontario and Scotland. Key informants from Ireland highlighted that protected funding had enabled investment in new radiotherapy facilities (*“despite through all the financial crisis of 2008-2009, there was still a wall around the cancer money”* [R10 Ireland]), although the financial crisis had meant delays in implementing the programme in parts of the country^43^ (*“a backlog just to catch up on some of the original plans”* [R16 Ireland]). As with the challenges reported with diagnostic equipment, lack of capital funding was reported to have undermined radiotherapy capacity in England and New Zealand, (“*There’s not enough machines” *[R26 New Zealand]).

 Yet, even where the physical infrastructure was in place, challenges to providing optimal treatment were noted (“*the trouble is, like all these things, you solve one problem and another bottleneck pops up as an issue.” *[R73 Scotland]). Respondents from several jurisdictions highlighted the interconnectedness of the cancer and wider health systems (*“all this other stuff going on”*), alongside adequate staffing, which can undermine an otherwise ‘reasonably well-balanced’ infrastructure:

*“So, in some tumour sites there are not enough theatre lists to meet […] demand but we would have surgeons to staff them but in other areas we just don’t have the surgical workforce either to deliver the volume. […] So, we have a challenging pathway in urology services where we simply don’t have enough surgeons to meet the cancer workload but equally if we had more surgeons, we wouldn’t have enough theatre lists” *[R35 Northern Ireland].

 Access to intensive care unit (ICU) beds and surgical capacity within hospitals were frequently mentioned by respondents. They noted that advancements in surgical techniques had made it possible to operate on some cancer patients who would previously not have been considered for surgery, for example due to co-existing conditions, but this had also increased the demand for ICU beds. One key informant from Denmark pointed to intense competition for these beds, with their perceived availability (or lack thereof) being considered when deciding on treatment of certain patients. Respondents from Ireland and Scotland further pointed to the pressures created by an ageing population, with increasing numbers of frail older patients occupying ICU beds (and acute care beds more widely, as noted by respondents from Ontario), thereby ‘squeezing’ out patients who need ICU admission following planned cancer surgery (*“those knock-on effects, they try not to cancel cancer patients, but inevitably [they have to]” *[R73 Scotland]). In England, most respondents expressed concerns about the pressures on NHS hospitals that had built up over recent years, and the lack of spare capacity, which will inevitably affect patient outcomes:

*“Last year, in the winter bed crisis, [location] ended up cancelling […] major cancer surgery, mostly due to lack of critical care bed space. But then, that was in the midst of a complex flu outbreak and there would always be those sort of bottlenecks. […] usually we manage to get it through, but that will happen, but having spare capacity – we try and run our hospitals and somewhere between 85% and 92% occupancy. And we’re never there, we’re always at much higher – 95%, 96%, some of them 101%” *[R65 England].

 Pressures such as those described above were not reported everywhere, with respondents from Australia and Norway viewing surgical capacity, in the form of operating theatres and bed numbers, as sufficient.

###  Workforce Along the Cancer Care Pathway

 Staff shortages were commonly cited as preventing countries from making as much progress in cancer outcomes as elsewhere, with consequences all along the care pathway. Respondents attributed these shortages to a range of factors, from training insufficient numbers and an ageing workforce, with retirement of existing staff, to workforce migration and difficulties recruiting and retaining specialists in rural or remote settings, although the relative importance of these varied within and across jurisdictions.

 A common challenge reported across the board was that of meeting the increasing and increasingly complex demand for medical oncology services (*“the input you need to keep the patient on treatment and there aren’t enough bodies to do that. There’s not enough bodies to do the demand”* [R63 England]). This was, as expected, greatest where there were shortages of these specialists, as in Wales, but even those jurisdictions that had invested heavily in the medical oncology workforce, including Australia, Ireland, Manitoba and Norway, reported difficulties in keeping up with increasing demand vis-à-vis therapeutic advances:

*“The cracks are starting to appear in it because of the expense and complexity that’s coming on everywhere now, and the expectations and the immunotherapy, and patients are no longer dying, they’re living so our clinics are getting bigger and bigger and bigger… We’re currently suffering a bit of angst with low manpower numbers, and we’re having to put patients on waiting lists. We have never done that before” *[R55 Western Australia].

####  The Wider Workforce

 However, even where the medical oncology workforce was considered adequate, specialists depend on other medical and nursing staff to provide the multidisciplinary care that, as many respondents noted, are needed to deliver high-quality care to cancer patients and that, ultimately, will improve survival.

*“And that’s the real thing. Now the rate-limiting steps vary from one part of the United Kingdom to another. So, in some places it’s actually a shortage of nurses to look after patients on the ward, in other places it’s the number of nurses that you have available in operating theatres. In others its lack of intensive care staff, so if you look at, say, nursing shortages, you know, in one institution it could be one of those groups, in another institution it could be another” *[R79 England].

 Staff shortages were reported as particularly challenging by respondents from smaller jurisdictions and those with large rural or remote areas. Here, the main issue was recruitment and retention in the context of an increasingly global market. For instance, participants from Ireland noted a history of health professionals trained there migrating elsewhere, in particular following the 2007/2008 financial crisis, with one respondent pointing to the impact this had on delivering radiation therapy and, thus, cancer outcomes:

*“[W] e’ve had no difficult[y] in attracting high-quality trainees, and we train them and most of them leave the country and don’t come back. So, we are operating a very effective radiology training system for Canada, the United States and the UK, and a bit of Australia now as well” *[R25 Ireland].

 Within-country competition for nurses and specialists was cited as an added challenge for Canada and the United Kingdom. For example, respondents from Ontario highlighted a shortage of radiotherapy staff, as locally trained physicians migrated to other Canadian provinces. Participants in other, poorer provinces reported how physicians were moving to wealthier ones. Respondents from Northern Ireland, Scotland and Wales pointed to the challenges the wider ‘UK-market’ for health professionals posed for them:

*“I mean let’s take a figure, if you take, I don’t know, 5 physicists in each cancer centre, maybe we’ve got 25 or 30 physicists across Scotland, if 4 of them leave [for England] that’s 20% of our workforce gone. If you’ve got 500 in England and 40 leave, it won’t even scratch the surface. So, we are more vulnerable to movement of whole people” *[R77 Scotland].

 Many countries have long faced major challenges in staffing rural and remote communities, with contemporary examples reported by participants from Canada, Denmark, Ireland, Northern Ireland, Scotland, and Wales. For instance, a Nova Scotian participant recounted that a small community in the province had managed to obtain a radiotherapy machine, but not the staff to operate it (*“They just don’t want to go there”* [R14 Nova Scotia]). Participants from several jurisdictions described how regionalisation of services and the use of telehealth could overcome some of these challenges, providing cancer services closer to people’s homes, but this could not overcome all the challenges of attracting and retaining staff.

## Discussion

###  Main Finding

 Study participants from 7 high-income countries shared their perspectives of how the capacity of health systems, in the form of health workers and physical infrastructure, here facilities and equipment, had impacted on the ability to provide timely and effective care. Their observations are, in themselves, not surprising, and illustrated by, for example, empirical evidence of variation in access to cancer diagnosis and treatment, although the clinical impact in terms of survival outcomes is often not clear.^47,48^ However, the added insights offered by stakeholders interviewed for this study are the interdependencies between different parts within the cancer system, and the cancer and wider health system within which it sits. Many public and political discourses about cancer outcomes are dominated by much narrower issues, such as access to a new drug that might benefit a handful of patients and, even then, for only a short time.^49^ It is easy to overlook the bigger picture, whereby the ability to improve outcomes, through faster diagnosis and better treatment, depends on the people who provide the care and the tools they have to do so. Crucially, this includes resources in the broader health system and not just those elements we think of as most intimately involved in cancer care.

 The 7 countries studied have achieved very different survival rates and have progressed at different speeds (see Supplementary file 1).^2^ Yet they all have health systems that are, in many respects, comparable. For this reason, they offer a rich natural laboratory from which to learn. Patients with cancer embark on a journey through different diagnostic and treatment processes, navigating a complex health system in which there are both barriers to slow them and facilitators to speed them on their way.^32^ Importantly, a barrier at one point can have consequences that affect subsequent stages in the journey, as clearly highlighted by participants in this study.

 Yet, there are challenges in drawing lessons that can be generalised. Health systems are, like all human systems, complex and adaptive. A change in one aspect can have consequences in others, with both positive and negative feedback loops. As interview participants noted, changes in service delivery and advances in diagnosis and treatment were interlinked. Thus, while centralisation of surgical provision was seen widely as good, to achieve all the benefits it was necessary to implement wider service reconfigurations, including the introduction of multi-disciplinary teams. Moreover, specific compensatory mechanisms may be needed where facilities serve remote rural areas.

###  Comparison With Existing Literature

 The importance of context is apparent from the empirical literature. At one level, there is evidence to link a broadly-based programme of reform to improved outcomes, as has, for example, been shown for the English national cancer plan.^50^ There is also systematic review evidence of improvements in survival for certain cancers treated in specialised centres compared to those receiving treatment elsewhere, such as ovarian and colorectal cancer.^51-53^ However it is much more difficult to *directly* link service change to cancer outcomes at the *system* level. For example, Varagunam et al studied the centralisation of oesophago-gastric cancer services in England from the early 2000s.^54^ This found that the number of NHS trusts performing surgical resections more than halved over a 10-year period while surgical volume within trusts doubled. Postoperative mortality also fell, but only a small part of this decline was explained by NHS trust volume. Similarly, a study of pancreatic cancer in Denmark reported that surgery had been centralised from 12 performing units in 2001 to 4 by the mid-2010s. While volume had increased in all 4 units over time, 2 of the centres performed about 80%; importantly there was no clear relationship between volume and one-year mortality post-surgery, although numbers were small.^55^

 A related concern is the uneven implementation of centralisation of cancer surgery across jurisdictions as has been noted for ovarian cancer in England.^56^ Evidence from other service areas, such as stroke services,^57^ highlights that the implementation of national guidance on service reconfiguration including centralisation is rarely implemented uniformly across local systems, reflecting local capacity, resources and commitment. It was this latter point that our interview participants kept returning to, emphasising the capacity of the health system to respond, but to do so in an integrated fashion. As was noted, there is no point in acquiring an expensive scanner if there are no staff to operate it. The majority of jurisdictions included in this study have invested in physical infrastructure for diagnosis and testing as well as in particular groups of health workers, such as medical oncologists and radiologists. Yet, the pace of investment has frequently not (or only just) caught up with pace of patient demand and advances in treatment, an issue that has come to the fore in the more recent period in most jurisdictions. The only exceptions were Denmark and Norway, and, more recently, Australia, but even here rising demand, combined with the availability of innovative new treatments, is beginning to outstrip capacity, both in terms of infrastructure and workforce.

 Finally, although we focused on discrete jurisdictions, several participants emphasised the global nature of healthcare, with migration of skilled health workers within and between countries posing significant challenges. It seems counterintuitive to train people with marketable skills if the system fails to provide the incentives necessary to retain them. Worldwide, there is a shortage of medical/clinical oncologists, many of whom report high clinical volumes, insufficient time for continuing professional development, a lack of support from other staff, and limited access to newer treatments.^22,23,58^ This requires a global solution.

###  Strengths and Limitations

 While we were able to draw on a wide range of key informants in the cancer care and wider health systems fields in the 7 high-income countries, some jurisdictions and organisations were better represented than others (Table), and we might lack perspectives where the number of interview participants was small. Likewise, interviews were carried out in English language only, including in Denmark and Norway. It is possible that we missed key informants in these countries who did not feel confident speaking English. Nevertheless, we interviewed people in senior roles, and we observed that respondents from a given jurisdiction were typically in agreement about key observations with very few conflicting positions. Thus, we feel confident that we have captured key perspectives. Importantly, our unit of investigation was the jurisdiction but we are aware of within-jurisdiction variation in access to cancer diagnosis and treatment or of treatment patterns,^47,59,60^ an issue that was commented on by many study participants, too. Indeed, variation within jurisdictions may be greater than variation between jurisdictions. This highlights the need for studies that explore sets of local ‘cancer systems’ in the context of the wider health system in order to better understand the impacts of differences in diagnostic and treatment patterns and their impacts on cancer patient outcomes.

## Conclusion

 This study highlights the complexity of factors that contribute to observed improvements in cancer survival across countries and over time. Although the relative importance of different factors will vary across settings, it was possible to plausibly link investment resources, that is, staff, infrastructure and equipment for diagnosis and treatment, and processes, in particular service consolidation and surgical specialisation, with improved quality of care. It confirms observations from elsewhere that have emphasised the importance of primary care and timely diagnosis for cancer survival although the treatment part of the cancer care journey was equally key to observed improvements. Continued improvement in cancer outcomes will require sustained strategic investment in plans to deliver and maintain the workforce engaged in cancer care and in the infrastructure on which they depend. However, strategic plans must recognise that systems for cancer care do not work in isolation from the rest of the health system and a whole systems approach is essential if we are to improve outcomes for an ageing, increasingly multimorbid population.

## Acknowledgements

 The authors would like to thank all key informants for giving their time to be interviewed for this study. We further thank Lucie Hooper, Samantha Harrison, Charles Norell, Shanta Keshwala and Charlotte Lynch of Cancer Research UK for managing the programme. The ICBP Clinical Committees for their advice. The ICBP Academic Reference Group for providing independent peer review and advice, in particular: Nancy L. Keating, MD, MPH. Harvard Medical School; Professor Jane Young. University of Sydney; Dr. Stuart Peacock. Co-Director of the Canadian Centre for Applied Research in Cancer Control and Head of Cancer Control Research at BC Cancer; Professor Kathy Pritchard-Jones. University College London. Finally, we are very grateful to the ICBP Programme Board for their oversight and direction.

## Ethical issues

 Ethical approval was granted by the Observational/Interventions Research Ethics Committee at the London School of Hygiene & Tropical Medicine, London, UK (LSHTM Ethics Ref: 15169).

## Competing interests

 Authors declare that they have no competing interests.

## Authors’ contributions

 MS drafted the initial manuscript, EN developed the manuscript further and led on the final draft; MM and MMcK reviewed the draft and contributed to editing; MS, MM and EN were involved in data collection and analysis; EN and MMcK conceptualised the overall study; EN is guarantor.

## Funding

 The ICBP is funded by the Canadian Partnership Against Cancer; Cancer Council Victoria; Cancer Institute New South Wales; Cancer Research UK; Danish Cancer Society; National Cancer Registry Ireland; The Cancer Society of New Zealand; NHS England; Norwegian Cancer Society; Public Health Agency Northern Ireland on behalf of the Northern Ireland Cancer Registry; DG Health and Social Care, Scottish Government; Western Australia Department of Health; Public Health Wales NHS Trust.

## Endnotes

 [1] According to data from the International Atomic Energy Agency, in 2018/2019 Denmark had 9.1 LINACs per 1 million population compared to 9/1m in Australia, 8/1m in Ireland, 7.9/1m in Norway, 7.5/1m in Canada, 5.7/1m in New Zealand and 5.2/1m in the United Kingdom. However, data are difficult to compare as some countries include machines in the private sector (eg, Ireland), while others do not (eg, New Zealand).

## Supplementary files


Supplementary file 1. Interview Topic Guide.
