# Supplementary material for: "There’s Not Enough Bodies to Do the Demand": An Exploration of Key Stakeholder Views on the Role of Health Service Capacity in Shaping Cancer Outcomes in 7 International Cancer Benchmarking Partnership Countries
Source: Int J Health Policy Manag. 2020 Dec 22;11(7):1024–34. doi: 10.34172/ijhpm.2020.254 (PMC9808162; doi:10.34172/ijhpm.2020.254)
Supplement: Supplementary file 1 — Interview Topic Guide. [file ijhpm-11-1024-s001.pdf]

Supplementary file 1. Interview Topic Guide

Understanding international variation in cancer outcomes: an analysis of patient pathways and the role of health systems

Introduction

- Could you briefly introduce yourself and describe your current role and responsibilities?

1. **The role/importance of cancer services in the wider health system**

- How would you describe the importance that is given to cancer services compared to other policy areas (e.g. diseases such as cardiovascular disease, diabetes, or populations, e.g. older people) in *[jurisdiction]*?

  For example:
  - *importance in terms of funding allocated; identified national/jurisdictional priority in form of cancer plan/strategy (or part of wider non-communicable diseases strategy?); etc.*
  - *has this changed over time and in what way?*

2. **Variation in cancer survival and trends over time**
   - Please have a look at Figure 1.
   - Focusing on [jurisdiction] first: How would you explain the observed variation in trends over time for different cancers?

     For example: role of centralisation; cancer strategies/plans; performance targets; etc.

   - Looking now at *[jurisdiction]* in comparison: What do you believe are the key 'health system' factors that could explain the relative performance compared to these others?

     For example: organisation of cancer services, workforce, funding, infrastructure, etc.

3. **The cancer patient journey**
   - Please have a look at Figure 2 (the patient journey).
   - Where do you see the key 'break points' or bottlenecks along the cancer patient journey in [jurisdiction]that may impact on survival?
     - *Human resources: workforce challenges*
     - *Physical resources: access to diagnostic equipment; intensive care beds; radiotherapy; chemotherapy; funding shortages*
     - *Knowledge resources: standardised referral pathways; access to guidelines; interconnected information systems; quality assurance*

   - How widespread do you think these problems are *[jurisdiction]*?

For example: variation in access to high-quality treatment: rural vs. urban; impact of deprivation; other?

- Where have key achievements been made over the past few years that have overcome (some of these) break points and how?

4. Policy context

- Reflecting again on the priority (or not) given to cancer services *[jurisdiction]*: To what degree do you think this has impacted on investment into the service and how?

  For example: workforce, infrastructure, other capacity

- What policy instruments are used and, in your experience, how effective have they been?

  For example: Benchmarking; targets; incentives; regulation

- What do you believe are the main issues and challenges facing *[jurisdiction]* at present?

5. The future

- How do you see cancer services in *[jurisdiction]* evolving in the future?
  - *What are the key opportunities?*
  - *What are the key barriers (e.g. austerity/financial pressures; workforce – recruitment, retention, training, cuts; infrastructure)*

- Are there any other issues you would like to raise that you believe are likely to explain variation in outcomes across countries as far as capacity is concerned?

Could you recommend other people in *[jurisdiction]* you think we should talk to?

Figure 1. Trends in survival from selected cancers, 2000-2014 (SURVMARK2 data[1])

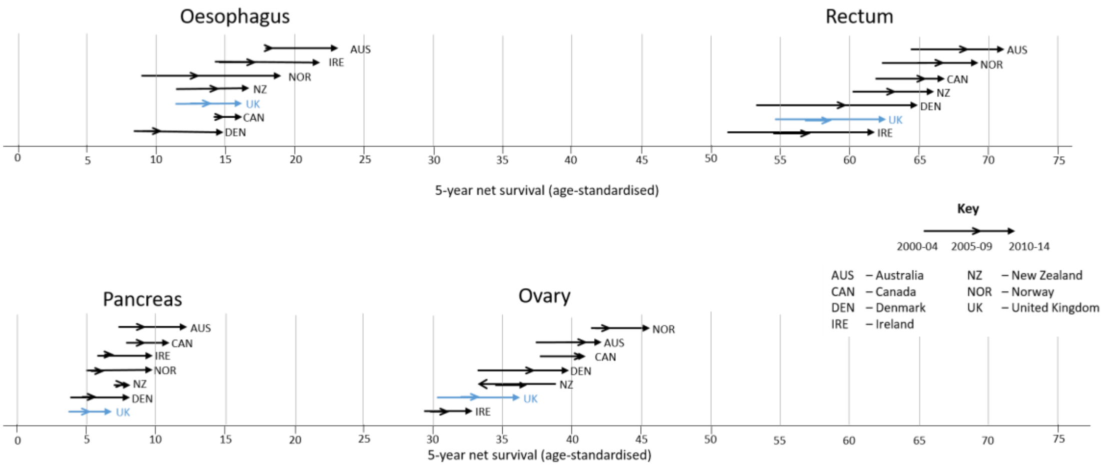

Figure 2. The cancer patient journey: a simplified model

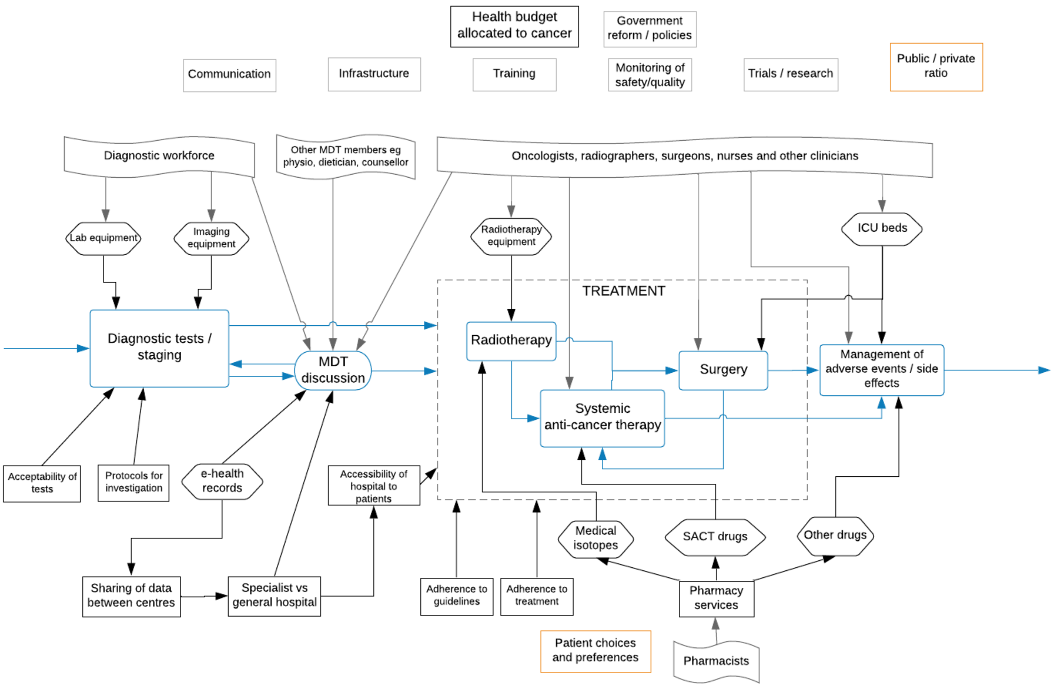

[1] Arnold M, Rutherford M, et al. *Lancet Oncol.* 2019 Nov;20(11):1493-1505
